# Editorial: Corporate entrepreneurs and psychological implications

**DOI:** 10.3389/fpsyg.2024.1380653

**Published:** 2024-03-04

**Authors:** Aidin Salamzadeh, Vitor Braga, Mirjana Radovic-Markovic

**Affiliations:** ^1^Faculty of Management, University of Tehran, Tehran, Iran; ^2^CIICESI, ESTG, Politécnico do Porto, Porto, Portugal; ^3^Faculty of Economics and Engineering Management, University Business Academy, Novi Sad, Serbia

**Keywords:** entrepreneur, corporate entrepreneur, intrapreneur, psychology, implications

Corporate entrepreneurs/intrapreneurs are called organizational heroes who go through an entrepreneurial journey within a typical organizational setting. Then, they must struggle to exist in a VUCA business environment and survive in an organizational context. During such a journey, they experience various emotional and psychological issues, such as anger, aggression, and depression, as well as many mental problems. Although the extant literature on the psychological aspects of entrepreneurship is rich, a few studies have already focused on corporate entrepreneurs/intrapreneurs. Therefore, this Research Topic includes four studies that shed light on different aspects of the issue in question.

In a seminal article, Li et al. focused on how the COVID-19 pandemic affected entrepreneurs anxiety. They explored the impact of entrepreneurs' threat perception and their performance pressure among 168 entrepreneurs of entrepreneurial firms. Their findings revealed that the pandemic positively affected entrepreneur anxiety, and the threat perception mediated the relationship between the pandemic and entrepreneur anxiety. Besides, it was highlighted that this impact could become stronger considering entrepreneur performance pressure, while the entrepreneur performance pressure weakened it. This study is unique in terms of its focus on corporate entrepreneurs' psychological and mental issues while facing (global) crises.

The study by Ali et al. explored the effect of corporate social responsibility (CSR) on firm reputation and organizational citizenship behavior (OCB). They also considered the mediating role of organic organizational cultures to deepen their findings. This study focused on two critical phenomena, i.e., organizational citizenship behavior and CSR, which are integral to any successful corporate entrepreneurial activity. They gathered data from 360 employees of selected enterprises in Pakistan. The authors found that CSR affects both firm reputation and OCB. Besides, their findings revealed that organic organizational culture mediates the relationship between CSR and firm reputation, and between CSR and OCB.

Tisu et al. explored the antecedents and mediators in the relationship between entrepreneurial wellbeing (EWB) and business performance. They studied 217 Romanian entrepreneurs to examine their psychological capital and its impact on the performance of their firms. Their model clarified the relationship between entrepreneurs' roles at work and home and their connection with EWB and business performance. They explored various relevant variables, including psychological capital, work engagement, work-life balance, entrepreneurial satisfaction, mental health, and performance. They believe that interventions according to this model “*can psychologically prepare entrepreneurs to be successful in their entrepreneurial endeavors*”.

Finally, Van Gelderen proposed an experiential learning technique to develop students' entrepreneurial mindset and competencies. Besides, he used this learning format to investigate the elements that could promote their competencies and affect their mindsets. He explored the experiences of a hundred and eighty-eight participants in six courses from five countries who participated in this experiential learning journey. According to his findings, “*Learning occurs when participants leave their comfort zone and have experiences that surprise them, leading to novel realizations. Key to learning is the element of surprise*”. This finding is a vital point to be considered by corporate entrepreneurs, as they could convey their experiences to potential corporate entrepreneurs by letting them know about experiences that have surprised them.

In sum, the studies mentioned above contributed to the field of corporate entrepreneurship by providing diverse insights into the psychological implications of entrepreneurial journeys inside firms.

## Author contributions

AS: Conceptualization, Supervision, Writing—original draft. VB: Writing—original draft. MR-M: Writing—original draft.

